# Pan-Immune-Inflammation Value Could Be a New Marker to Predict Amyloidosis and Disease Severity in Familial Mediterranean Fever

**DOI:** 10.3390/diagnostics14060634

**Published:** 2024-03-16

**Authors:** Tuğba Ocak, Ahmet Görünen, Belkıs Nihan Coşkun, Burcu Yağız, Sebnem Ozemri Sağ, Gökhan Ocakoğlu, Ediz Dalkılıç, Yavuz Pehlivan

**Affiliations:** 1 Department of Rheumatology, Uludag University Faculty of Medicine, Nilufer 16059, Bursa, Turkey; belkisnihanseniz@hotmail.com (B.N.C.); burcuyilmaz_84@hotmail.com (B.Y.); edizinci@hotmail.com (E.D.); drypehlivan@gmail.com (Y.P.); 2 Department of Internal Medicine, Uludag University Faculty of Medicine, Nilufer 16059, Bursa, Turkey; ahmetgorunen@uludag.edu.tr; 3 Department of Medical Genetics, Uludag University Faculty of Medicine, Nilufer 16059, Bursa, Turkey; ozemri@uludag.edu.tr; 4 Department of Biostatistics, Uludag University Faculty of Medicine, Nilufer 16059, Bursa, Turkey; gocakoglu@gmail.com

**Keywords:** pan-immune-inflammation value, familial Mediterranean fever, amyloidosis, moderate-to-severe disease

## Abstract

Familial Mediterranean fever (FMF) is characterized by recurrent episodes of fever and serositis. Blood-based biomarkers determined in FMF patients during attack-free periods could be used to predict the risk of amyloidosis and the severity of the disease. The recently defined pan-immune-inflammation value (PIV) comprises four distinct subsets of blood cells and serves as an easily accessible and cost-effective marker. The objective of this study was to assess the role of PIV in predicting amyloidosis and moderate-to-severe disease. Clinical characteristics and laboratory values during the attack-free period were retrospectively analyzed in 321 patients over 18 years of age diagnosed with familial Mediterranean fever (FMF). In our tertiary adult rheumatology outpatient clinic, disease severity and laboratory markers were evaluated during the first attack-free interval. At baseline, patients with amyloidosis were excluded. Patients were categorized based on the presence of amyloidosis and the severity of the disease. When focusing on amyloidosis in receiver operating characteristic (ROC) analysis, optimal cut-off values for pan-immune-inflammation value (PIV), neutrophil-to-lymphocyte ratio (NLR), and platelet-to-lymphocyte ratio were determined as ≥518.1, ≥2.3, and ≥127.2, respectively. In multivariate analysis, PIV, C-reactive protein (CRP), and the presence of the M694V homozygous mutation emerged as independent risk factors for both amyloidosis and moderate-to-severe disease. Additionally, NLR was identified as an independent risk factor for amyloidosis, while red blood cell distribution width was associated with moderate-to-severe disease. In patients with FMF, especially in the presence of the M694V homozygous mutation, CRP and PIV may be useful in predicting both amyloidosis and moderate-to-severe disease.

## 1. Introduction

Familial Mediterranean fever (FMF) is the most common monogenic hereditary autoinflammatory disease characterized by recurrent episodes of serositis and fever [1]. It is more common among Turks, Arabs, non-Ashkenazi Jews, and Armenians [2]. FMF is caused by mutations in the Mediterranean fever gene (MEFV), which codes for the pyrin protein, which plays an important role in the regulation of inflammatory processes [3]. Mutated pyrin activates caspase 1 and causes excessive secretion of interleukin-1 beta (IL-1β). Elevated levels of IL-1, accompanied by high levels of cytokines such as IL-6 and tumor necrosis factor-alpha (TNF-α), lead to an increase in leukocytes and acute-phase reactants as well as systemic inflammation [4].

Attacks usually last 1–3 days, and leukocytes and acute-phase tests normalize during the attack-free period. Nevertheless, in some patients, leukocytes and acute-phase reactants remain elevated even in the absence of an attack, which is considered subclinical inflammation [5]. FMF-related complications may increase the risk of morbidity and mortality [6]. Amyloidosis is the most serious complication of FMF [7]. Subclinical inflammation is thought to be an important factor in the development of amyloidosis, and it is important to recognize this condition in FMF patients [8]. In the attack-free period, acute-phase reactants can be used to monitor subclinical inflammation and predict the risk of amyloidosis and severe disease [9]. Complete blood count tests are inexpensive and easily accessible. Parameters such as pan-immune-inflammation value (PIV), neutrophil-to-lymphocyte ratio (NLR) and platelet-to-lymphocyte ratio (PLR) determined by the complete blood count test can be used as markers for systemic inflammation. There are few studies using hematologic indices to predict amyloidosis and disease severity in adult FMF patients, and these studies were performed using NLR and PLR [10,11,12,13,14,15,16].

Fuca et al. showed in 2020 that PIV was better than NLR in predicting the survival of patients with advanced colorectal cancer [17]. PIV is a recently developed biomarker that is calculated by multiplying the neutrophil count by the platelet and monocyte counts and then dividing the result by the lymphocyte count [18]. In a study conducted in patients with antineutrophil cytoplasmic antibody (ANCA)-associated vasculitis (AAV), survival was found to be lower in patients with high levels of PIV at the time of diagnosis [19]. To our knowledge, there is no study evaluating the PIV biomarker in the prediction of amyloidosis and disease severity. In our study, we aimed to evaluate the role of hematologic biomarkers such as NLR and PLR, especially PIV, in addition to the known clinical and laboratory risk factors in the prediction of amyloidosis and moderate-to-severe disease.

## 2. Materials and Methods

### 2.1. Study Population

Our retrospective study analyzed electronic records from 438 patients diagnosed with FMF according to the Tel-Hashomer criteria [20]. These patients were followed up as outpatients at Bursa Uludag University Faculty of Medicine, Department of Rheumatology, between January 2010 and April 2022.

All patients included in this study were regularly treated with colchicine and were over 18 years of age. At baseline, patients with solid organ malignancies and hematologic diseases, acute and chronic infections, primary or secondary immunodeficiency, a glomerular filtration rate <60 mL/min/1.73 m^2^ according to the Kidney Disease Improving Global Outcome Group (KDIGO) criteria [21], additional rheumatologic diseases, patients receiving biologic therapy, pregnant or breastfeeding patients, patients with amyloidosis, and patients with missing data were excluded. Following these criteria, this study was conducted on a total of 321 patients.

### 2.2. Study Design and Data Collection

Demographic characteristics such as age, sex, and comorbidities (diabetes mellitus, hypertension, cardiovascular disease, cerebrovascular disease, hypothyroidism, hyperthyroidism, chronic obstructive pulmonary disease) were analyzed. The comorbidity index was used to measure the comorbidity of the patients [22]. Patient age, history of appendectomy, amyloidosis status, age at FMF diagnosis, family history of FMF, family history of amyloidosis, clinical symptoms (fever, abdominal pain, chest pain, arthritis/arthralgia, myalgia, erysipelas-like erythema), and the presence of hepatomegaly and splenomegaly were assessed. All patients with FMF and nephrotic syndrome (proteinuria > 3.5 g/24 h) underwent a kidney biopsy, while other patients with lower proteinuria values underwent a biopsy of the rectum, gingiva, or duodenum. Disease severity was recorded. The severity of FMF was assessed using the International Severity Scoring System (ISSF). The ISSF ranges from 0 to 10 and classifies severity as severe disease ≥ 6, moderate disease (3–5), and mild disease (≤2) [23].

Disease severity and laboratory parameters were assessed during the first attack-free period at a visit to the adult rheumatology outpatient clinic. The attack-free period was defined as at least 2 weeks after the end of the attack. We examined the erythrocyte sedimentation rate (ESR), C-reactive protein (CRP), and a complete blood count, including white blood cells (WBCs), neutrophils, lymphocytes, monocytes, eosinophils, basophils, platelets, hemoglobin (Hb), hematocrit (Htc), red blood cell count, mean corpuscular volume (MCV), mean corpuscular hemoglobin (MCH), mean corpuscular hemoglobin concentration (MCHC), red blood cell distribution width (RDW), and mean platelet volume (MPV). Additionally, MEFV mutations were investigated. NLR was calculated as the ratio of neutrophil count (10^3^/mL) to lymphocyte count (10^3^/mL) and PLR as the ratio of platelet count (10^3^/mL) to lymphocyte count (10^3^/mL) in the attack-free period. PIV was calculated by multiplying the neutrophil count (10^3^/mL) by the platelet count (10^3^/mL) and the monocyte count (10^3^/mL) and then dividing the result by the lymphocyte count (10^3^/mL).

We initially divided the patients into two groups according to the presence of amyloidosis to test our hypothesis. Then, we compared clinical and laboratory features between the groups. Additionally, we classified patients as mild disease (ISSF score ≤ 2) and moderate-to-severe disease (ISSF score > 3) [23]. Subsequently, we compared clinical and laboratory features between patients with mild and moderate–severe disease. In addition to indices such as NLR, PLR, and PIV, previously studied complete blood count parameters associated with amyloidosis and disease severity, acute-phase tests such as ESR and CRP, known risk factors for amyloidosis and severe disease such as the male sex, age at disease diagnosis, family history of amyloidosis, Charlson comorbidity index, and positivity of the homozygous mutation M694V, as well as the presence of a family history of amyloidosis as an additional risk factor for amyloidosis, were included in further analysis [10,24,25,26,27,28].

### 2.3. Statistical Analysis

Statistical analysis was conducted using SPSS (Statistical Package for Social Sciences) version 26.0. The normality of variables was assessed using the Shapiro–Wilk and Kolmogorov–Smirnov tests. Quantitative data are expressed as mean ± standard deviation for normal distribution and as median (interquartile range, IQR) for non-normal distribution. The Mann–Whitney U test and independent sample *t*-test were employed for quantitative variables, while the chi-square test was used for qualitative variables. The optimal cut-off points for NLR, PLR, and PIV were determined by receiver operating characteristic (ROC) analysis with amyloidosis as the point of interest. Univariate and multivariate logistic regression analyses were performed to identify predictors of amyloidosis and moderate-to-severe disease. Multivariate logistic regression analysis (forward LR) was used for variables with a *p*-value below 0.25 in the univariate analysis. *p*-value < 0.05 was considered statistically significant. The net reclassification improvement (NRI) and the integrated discrimination index (IDI) were calculated to evaluate the improvement of the discrimination ability of the baseline model by adding NLR and PIV for amyloidosis.

## 3. Results

This study included a total of 321 patients, and their general characteristics are summarized in Table 1. Of the patients, 196 (61.0) were female. The patients’ median age was 26.2 years (18.0–62.3). The most common symptom associated with FMF was abdominal pain, in 303 (94.3%) patients. The most frequently observed mutation type was M694V homozygous, present in 82 patients (25.5%).

### 3.1. Association between Amyloidosis and Clinical and Laboratory Features

Amyloidosis detected by tissue biopsy was diagnosed in 27 (8.4%) patients. Amyloidosis was diagnosed in 22 patients through kidney biopsy, in 3 through rectal biopsy, in 1 through duodenal biopsy, and in 1 through gingival biopsy. The relationship between amyloidosis and clinical and laboratory features is shown in Table 2. The male sex ratio, creatinine, WBC, neutrophils, monocytes, RDW, platelets, NLR, PLR, PIV, sedimentation, CRP, and the rate of positivity for the homozygous mutation M694V were significantly higher in the group with amyloidosis (*p* < 0.001, *p* < 0.001, *p* < 0.001, *p* < 0.001, *p* < 0.001, *p* < 0.001, *p* < 0.001, *p* = 0.033, *p* < 0.001, *p* < 0.001, *p* < 0.001, *p* < 0.001, *p* < 0.001, *p* < 0.001, respectively). Conversely, lymphocyte count, hemoglobin (Hb), and hematocrit (Htc) values were significantly lower in patients with amyloidosis (*p* = 0.043, 0.035, 0.017, respectively). The values for the area under the curve, sensitivity, and specificity of the ROC analysis for amyloidosis are shown in Table 3. The cut-off values for PIV, NLR, and PLR were set at ≥518.1, ≥2.3, and ≥127.2 respectively. In the multivariate logistic regression analysis (Table 4), PIV (odds ratio [OR] 20.184; 95% confidence interval [CI], 4.267–95.467; *p* < 0.001), CRP (OR, 1.155; 95% CI, 1.048–1.272; *p* = 0.004), M694V homozygous mutation (OR, 4.314; 95% CI, 1.250–14.892; *p* = 0.021), and NLR (OR 12.549; 95% CI, 1.182–133.255; *p* = 0.036) were identified as independent risk factors. The improvement in predictive ability was further assessed using NRI and IDI. The analysis significantly improved using PIV (model-II) over NLR (model-I). The NRI was 0.263 with a 95% CI of [−0.078:0.575] and a *p* = 0.498, while the IDI was 0.055 with a 95% CI of [−0.022:0.135] and a *p* = 0.499. These results indicate that the statistical significance of these improvements was not established.

### 3.2. Association between Moderate-to-Severe Disease and Clinical and Laboratory Features

A total of 59 patients (18.3%) exhibited moderate-to-severe FMF. The relationship between moderate-to-severe disease and clinical and laboratory features is outlined in Table 5. WBCs, neutrophils, monocytes, RDW, platelets, NLR, PLR, PIV, sedimentation, CRP values, and M694V homozygous mutation positivity rate were significantly higher in the group with moderate-to-severe disease (*p* < 0.001, *p* < 0.001, *p* = 0.002, *p* < 0.001, *p* < 0.001, *p* < 0.001, *p* = 0.005, *p* < 0.001, *p* < 0.001, *p* < 0.001, *p* < 0.001, respectively). Hb, Htc, MCV, and MCH values were significantly lower in moderate-to-severe disease (*p* = 0.001, *p* = 0.001, *p* < 0.001, *p* < 0.001, *p* < 0.001, respectively). In the multivariate analysis performed with PIV, NLR, PLR, and cut-off values determined for clinical characteristics and amyloidosis, PIV (odds ratio [OR], 3.133; 95% confidence interval [CI], 1.406–6.978; *p* = 0.005), CRP (OR, 1.139; 95% CI, 1.059–1.225; *p* < 0.001), presence of the M694V homozygous mutation (OR, 3.714; 95% CI, 1.869–7.379; *p* < 0.001), and RDW (OR, 1.131; 95% CI, 1.007–1.270; *p* = 0.038) were identified as independent risk factors for moderate-to-severe disease (Table 6).

## 4. Discussion

In our study, the presence of the homozygous M694V mutation in FMF patients, along with PIV and CRP levels determined during the attack-free period, emerged as independent risk factors for predicting both amyloidosis and moderate-to-severe disease. Additionally, NLR for amyloidosis and RDW for moderate-to-severe disease were identified as independent risk factors.

Amyloidosis is the most serious complication of FMF and known risk factors include the male sex, the presence of the M694V mutation, early age at diagnosis, and the presence of amyloidosis in family members [24,25,26,29,30]. Another important risk factor is inflammation that persists despite colchicine treatment [8]. Various inflammatory markers, such as ESR, CRP, fibrinogen, serum amyloid A protein, and white blood cell levels, are utilized as acute-phase markers in FMF [31]. PIV is a novel blood-based biomarker encompassing immune cell subpopulations of neutrophils, platelets, monocytes, and lymphocytes in peripheral blood [19]. PIV has also been studied in various diseases under different names, such as the systemic immune-inflammation response index (SIIRI) and the aggregate index of systemic inflammation (AISI), and has been found to be associated with certain inflammatory conditions [32,33]. Studies on PIV have mainly been conducted in the field of oncology. There are only a few studies on PIV in the field of rheumatology. A study by Lee et al. examined PIV in patients diagnosed with AAV. The survival rate was lower in those who had higher PIV values at the time of diagnosis [19]. Additionally, Tutan et al. reported significantly elevated PIV in patients with active rheumatoid arthritis compared to those in remission [34]. As far as we know, previous studies have not investigated the relationship between PIV and amyloidosis and disease severity in FMF patients. In this study, PIV, evaluated during the attack-free period, was shown for the first time to be an independent risk factor for the prediction of amyloidosis and moderate-to-severe disease in FMF patients. PIV is a simple and easy-to-use biomarker that can be used in clinical practice to predict amyloidosis and moderate-to-severe disease.

CRP is an acute-phase protein of hepatic origin that rises in response to the release of IL-6 by macrophages and T cells and indicates the severity of inflammation. CRP increases in acute and chronic inflammation as an indicator of the acute-phase response [35]. Varan et al. have shown that high CRP levels in the attack-free period can be important for the development of amyloidosis [27]. The study by Tezcan et al. found that CRP levels measured during the attack-free period were high in moderate-to-severe disease [10]. In our study, CRP proved to be an independent risk factor for the prediction of both amyloidosis and moderate-to-severe disease.

To date, more than 300 MEFV mutations have been defined, and pathogenic mutations are most frequently found in exon 10. The most common of these mutations is M694V, with a frequency of 20–65% [36]. The presence of the M694V homozygous mutation is considered a risk factor for severe disease progression and amyloidosis [37,38]. In a multicenter study of Turkish patients by Kaşifoğlu et al., it was found that M694V homozygote positivity was significantly higher in patients with amyloidosis [26]. In a recent study conducted in pediatric patients in Turkey, it was shown that patients with the M694V homozygous mutation had more severe amyloidosis compared to other mutations, and this mutation was associated with disease severity [39]. In our study, M694V homozygote positivity also proved to be an independent risk factor for the prediction of moderate-to-severe disease and amyloidosis.

The NLR, which is determined by dividing the number of neutrophils by the number of lymphocytes, has been reported in various studies as an indicator of the systemic inflammatory response [31,40]. In a study by Uslu et al. investigating the NLR biomarker in FMF patients, it was found that NLR was statistically higher in patients with amyloidosis compared to patients without amyloidosis [31]. Ahsen et al. suggested that NLR could be used as an acute-phase marker in FMF patients [14]. Tezcan et al. showed that high NLR can predict amyloidosis [10]. However, other clinical and genetic risk factors for amyloidosis, such as sex, age at diagnosis, and the presence of M694V homozygotes were not assessed in the study by Tezcan et al. and no multivariate analysis was performed. In our study, NLR was found to be an independent risk factor for the prediction of amyloidosis. NLR assessment in FMF patients could be helpful in the prediction of amyloidosis. In our study, although PIV outperformed NLR in predicting disease severity, they were both found to be independent risk factors in the amyloidosis prediction model, and they were not superior to each other in further analysis.

RDW is a parameter of the complete blood count and reflects the variability of the size of the circulating erythrocytes. Changes such as inflammation and oxidative stress affect the erythroid series in the bone marrow and cause changes in RDW. Experimental studies have shown that inflammatory cytokines suppress erythrocyte development and lead to an increase in RDW as immature erythrocytes enter the bloodstream [41]. When analyzing studies on RDW in the field of rheumatology, it was suggested that RDW could be used as a potential marker to assess disease activity in Behçet’s disease, rheumatoid arthritis, and systemic lupus erythematosus [42,43,44]. There are few studies on RDW in FMF patients, and it has been shown that high RDW values may reflect subclinical inflammation in the attack-free period [5,12,15,45]. These studies did not investigate the relationship between RDW and disease severity. In our study, it was found that high RDW levels in the attack-free period in FMF patients may be an independent risk factor for predicting moderate-to-severe disease, and our study is the first study on this topic. Assessment of RDW in FMF patients may be helpful in predicting moderate-to-severe disease.

### Limitations

One of the limitations of our study is that it has a single-center retrospective design. Serum amyloid A level, like other inflammatory markers, is an important parameter for the prediction of amyloidosis. However, serum amyloid A level could not be evaluated because the serum amyloid A levels of most patients could not be assessed in the attack-free period. Early initiation of colchicine treatment in FMF patients leads to an improvement in seizures and the prevention of amyloidosis. Patients who had been receiving regular colchicine treatment were included in this study. However, the time interval between the age of onset of symptoms and the start of colchicine treatment could not be assessed.

## 5. Conclusions

Although the incidence of amyloidosis has decreased significantly in recent years due to the use of colchicine in FMF, amyloidosis is still the most serious complication. Patients should be followed up not only during the attack period but also in the attack-free period. The identification of new biomarkers, such as PIV, which can be used in daily practice and are inexpensive and easily accessible, could be helpful in predicting amyloidosis and moderate-to-severe disease. In particular, in the presence of M694V homozygotes, patients with elevated CRP and a PIV ≥ 518.1 cut-off in the attack-free period should be closely monitored for the risk of amyloidosis and moderate-to-severe disease.

## Figures and Tables

**Table 1:** Demographic and disease-related characteristics of FMF patients (*n*: 321).

Age (year) median (min., max.)	26.2 (18.0–62.3)
FMF diagnosis age (year) median (min., max.)	24.2 (1.8–61.7)
Amyloidosis diagnosis age median (min., max.)	29.2(18.4–55.9)
Sex (female/male) *n* (%)	196 (61.0)/125 (38.9)
Family history of FMF *n* (%)	150 (46.7)
Family history of amyloidosis due to FMF *n* (%)	4 (1.2)
Comorbidity score median (min., max.)	0 (0–2)
Fever *n* (%)	271 (84.4)
Abdominal pain *n* (%)	303 (94.3)
Chest pain *n* (%)	36 (11.2)
Arthralgia/Arthritis *n* (%)	159 (49.5)
Myalgia *n* (%)	34 (10.6)
Prolonged febrile myalgia *n* (%)	4 (1.2)
Erysipelas-like erythema *n* (%)	32 (10.0)
Hepatomegaly *n* (%)	24 (7.5)
Splenomegaly *n* (%)	23 (7.2)
ISSF score median (min., max.)	5 (2–6)
Amyloidosis *n* (%)	27 (8.4)
MEFV mutations type *n* (%)	
M694V homozygous *n* (%)	82 (25.5)
M694V heterozygous *n* (%)	54 (16.8)
M680I homozygous *n* (%)	10 (3.1)
M680I heterozygous *n* (%)	4 (1.3)
V726A homozygous *n* (%)	1 (0.3)
V726A heterozygous *n* (%)	5 (1.6)
E148Q homozygous *n* (%)	21 (6.5)
E148Q heterozygous *n* (%)	18 (5.6)
R202Q homozygous *n* (%)	15 (4.7)
R202Q heterozygous *n* (%)	11 (3.4)
P369S homozygous *n* (%)	3 (0.9)
P369S heterozygous *n* (%)	7 (2.2)
M694V/M680I *n* (%)	13 (4)
M694V/V726A *n* (%)	8 (2.5)
M680I/V726A *n* (%)	6 (1.9)
M680I/E148Q *n* (%)	1 (0.3)
M694V/R202Q *n* (%)	7 (2.2)
E148Q/M694V *n* (%)	3 (0.9)
M694V/R761H *n* (%)	1 (0.3)
E148Q/P369S *n* (%)	4 (1.3)
R202Q/J339F *n* (%)	4 (1.3)
R761H *n* (%)	6 (1.9)
No mutation	37 (11.5)

FMF = familial Mediterranean fever, ISSF = International Severity Score for FMF, MEFV = Mediterranean fever gene.

**Table 2:** Clinical and laboratory characteristics of FMF patients classified in terms of amyloidosis.

	Amyloidosis (−) *n* = 294	Amyloidosis (+) *n* = 27	*p*
FMF diagnosis age (year)	24.45 (1.80–61.70)	23.20 (4.30–53.70)	0.425 *^m^*
Sex (male/female)	106/188	19/8	**<0.001** *^x^*^2^
Family history of amyloidosis (present/absent)	3/291	1/26	0.298 *^fe^*
Creatinine (mg/dL)	0.70 (0.38–1.41)	1.04 (0.53–1.50)	**<0.001** *^m^*
WBC (10^3^/mL)	7.00 (4.11–16.6)	11.2 (6.7–17.6)	**<0.001** *^m^*
Neutrophil (10^3^/mL)	3.91 (1.67–14.80)	8.43 (4.54–14.1)	**<0.001** *^m^*
Lymphocyte (10^3^/mL)	2.24 (0.95–5.22)	1.90 (0.90–4.38)	**0.043** *^m^*
Monocyte (10^3^/mL)	0.51 (0.08–1.43)	0.71 (0.44–1.40)	**<0.001** *^m^*
Eosinophil (10^3^/mL)	0.13 (0.00–1.15)	0.10 (0.00–0.90)	0.449 *^m^*
Basophil (10^3^/mL)	0.05 (0.00–0.40)	0.05 (0.00–0.23)	0.579 *^m^*
RBC (%)	4.80 (0.36–6.05)	4.78 (3.31–6.02)	0.365 *^m^*
Hb (g/dL)	13.54 ± 1.57	12.86 ± 1.91	**0.035** *^t^*
Hct (%)	40.83 ± 4.32	38.67 ± 5.87	**0.017** *^t^*
MCV (fL)	84.70 (55.70–98.70)	84.00 (65.50–93.90)	0.293 *^m^*
MCH (pg)	28.10 (16.90–34.10)	27.60 (19.90–31.90)	0.435 *^m^*
MCHC (g/L)	33.20 (28.30–35.70)	33.10 (30.40–35.40)	0.899 *^m^*
RDW (%)	14.20 (10.00–34.00)	15.10 (10.00–21.60)	**0.033** *^m^*
PLT (10^3^/mL)	232.00 (120.00–515.00)	317.00 (185.00–812.00)	**<0.001** *^m^*
MPV (fL)	8.50 (5.10–15.60)	8.14 (6.22–12.20)	0.488 *^m^*
PDW (fL)	18.00 (1.70–24.00)	17.00 (10.70–118.40)	0.066 *^m^*
NLR	1.70 (0.62–13.83)	4.04 (2.10–9.72)	**<0.001** *^m^*
PLR	102.60 (34.67–257.86)	146.33 (73.12–352.17)	**<0.001** *^m^*
PIV	209.46 (33.81–2355.55)	1055.90 (310.70–3267.31)	**<0.001** *^m^*
Sedimentation (mm/h)	8.00 (2.00–54.00)	18.00 (4.00–81.00)	**<0.001** *^m^*
CRP (mg/L)	3.00 (1.00–25.20)	7.30 (2.00–40.90)	**<0.001** *^m^*
M694V homozygous (present/absent)	63/231	19/8	**<0.001** *^x^*^2^

FMF = familial Mediterranean fever, WBC = white blood cell, RBC = red blood cell, Hb = hemoglobin, Hct = hematocrit, MCV = mean corpuscular volume, MCH = mean corpuscular hemoglobin, MCHC = mean corpuscular hemoglobin concentration, RDW = red cell distribution width, PLT = platelets, MPV = mean platelet volume, PDW = platelet distribution width, NLR = neutrophil-to-lymphocyte ratio, PLR = platelet-to-lymphocyte ratio, PIV = pan-immune-inflammation value, CRP = C-reactive protein, *^m^* = Mann–Whitney test, *^x^*^2^ = Pearson’s Chi-squared test, *^fe^* = Fisher’s exact test, *^t^* = independent samples test. Statistically significant values are shown in bold.

**Table 3:** Receiver operating characteristic curve analyses for amyloidosis.

Curve	Cut-off Value	AUC	95% CI	*p* Value	Sensitivity (%)	Specifcity (%)
**PIV**	518.1	0.960	0.931–0.989	<0.001	88.9	92.2
**NLR**	2.3	0.928	0.894–0.961	<0.001	96.3	78.6
**PLR**	127.2	0.827	0.749–0.905	<0.001	81.5	75.5

AUC = area under the curve, CI = confidence interval, PIV = pan-immune-inflammation value, NLR = neutrophil-to-lymphocyte ratio, PLR = platelet-to-lymphocyte ratio.

**Table 4:** Univariate and multivariate logistic regression analysis of clinical and laboratory characteristics for prediction of amyloidosis.

Factor		Univariate Analysis			Multivariate Analysis		
		OR	95% CI	*p*	OR	95% CI	*p*
FMF diagnosis age	Years	0.991	0.959–1.023	0.580			
Sex (male)	Male (RC) vs. female	4.212	1.783–9.951	0.001	-	-	-
Comorbidity index	Score	2.007	0.892–4.518	0.092	-	-	-
Family history of amyloidosis(%)	Present (RC) vs. absent	3.731	0.375–37.154	0.262			
Hb	g/dL	0.766	0.597–0.984	0.037	-	-	-
MCH	pg	0.934	0.805–1.082	0.362			
MCHC	g/dL	1.009	0.707–1.439	0.962			
RDW	%	1.126	0.999–1.269	0.052	-	-	-
NLR	High (RC) vs. low	88.090	11.735–661.267	<0.001	**12.549**	**1.182–133.255**	**0.036**
PLR	High (RC) vs. low	13.567	4.957–37.128	<0.001	**-**	**-**	**-**
PIV	High (RC) vs. low	94.261	26.380–336.218	<0.001	**20.184**	**4.267–95.467**	**<0.001**
Sedimentation	mm/h	1.077	1.042–1.112	<0.001	**-**	**-**	**-**
CRP	mg/L	1.213	1.128–1.304	<0.001	**1.155**	**1.048–1.272**	**0.004**
M694V homozygous mutation	Present (RC) vs. absent	8.708	3.642–20.823	<0.001	**4.314**	**1.250–14.892**	**0.021**

FMF = familial Mediterranean fever, Hb = hemoglobin, MCH = mean corpuscular hemoglobin, MCHC = mean corpuscular hemoglobin concentration, RDW = red cell distribution width, NLR = neutrophil-to-lymphocyte ratio, PLR = platelet-to-lymphocyte ratio, PIV = pan-immune-inflammation-value, CRP = C-reactive protein, RC = reference category, OR = odds ratio, CI = confidence interval. Statistically significant values are shown in bold.

**Table 5:** Clinical and laboratory characteristics of FMF patients classified for disease severity.

	Mild Disease *n* = 262	Moderate–Severe Disease *n* = 59	*p*
FMF diagnosis age (year)	24.50 (1.80–61.70)	23.00 (2.30–53.70)	0.309 ^m^
Sex (male/female)	97/165	28/31	0.138 ^x2^
WBC (10^3^/mL)	6.97 (4.14–16.6)	9.02 (4.11–17.60)	**<0.001** ^m^
Neutrophil (10^3^/mL)	3.84 (1.83–14.80)	6.01 (1.67–14.10)	**<0.001** ^m^
Lymphocyte (10^3^/mL)	2.22 (1.05–5.19)	2.29 (0.90–5.22)	0.869 ^m^
Monocyte (10^3^/mL)	0.51 (0.08–1.43)	0.60 (0.23–1.16)	**0.002** ^m^
Eosinophil (10^3^/mL)	0.13 (0.00–1.15)	0.13 (0.00–0.40)	0.872 ^m^
Basophil (10^3^/mL)	0.05 (0.00–0.34)	0.06 (0.00–0.4)	0.356 ^m^
RBC (%)	4.80 (2.73–6.05)	4.80 (0.36–6.02)	0.960 ^m^
Hb (g/dL)	13.63 ± 1.52	12.86 ± 1.83	**0.001** ^t^
Hct (%)	41.03 ± 4.27	38.92 ± 5.09	**0.001** ^t^
MCV (fL)	85.00 (68.00–98.70)	83.20 (55.70–92.30)	**<0.001** ^m^
MCH (pg)	28.10 (16.90–34.10)	27.00 (18.00–31.90)	**<0.001** ^m^
MCHC (g/L)	33.20 (28.30–35.70)	33.10 (29.80–35.40)	0.405 ^m^
RDW (%)	14.00 (10–22.8)	15.60 (10.00–34.00)	**0.002** ^m^
PLT (10^3^/L)	231.90 (120.00–489.00)	266.00 (169.00–812.00)	**<0.001** ^m^
MPV (fL)	8.58 (5.10–15.60)	8.21 (5.36–14.10)	0.541 ^m^
PDW (fL)	17.85 (1.70–24.00)	18.00 (11.10–118.40)	0.513 ^m^
NLR	1.68 (0.62–13.83)	2.49 (0.65–9.72)	**<0.001** ^m^
PLR	103.54 (42.39–352.17)	120.77 (34.67–352.17)	**0.005** ^m^
PIV	207.31 (33.81–2355.55)	381.83 (60.99–3267.31)	**<0.001** ^m^
Sedimentation (mm/h)	8.00 (2.00–54.00)	12.00 (2.00–81.00)	**<0.001** ^m^
CRP (mg/L)	3.00 (1.00–25.20)	4.00 (2.00–40.90)	**<0.001** ^m^
M694V homozygous (present/absent)	49/213	33/26	**<0.001** ^x2^

FMF = familial Mediterranean fever, WBC = white blood cell, RBC = red blood cell, Hb = hemoglobin, Hct = hematocrit, MCV = mean corpuscular volume, MCH = mean corpuscular hemoglobin, MCHC = mean corpuscular hemoglobin concentration, RDW = red cell distribution width, PLT = platelets, MPV = mean platelet volume, PDW = platelet distribution width, NLR = neutrophil-to-lymphocyte ratio, PLR = platelet-to-lymphocyte ratio, PIV = pan-immune-inflammation value, CRP = C-reactive protein, ^m^ = Mann–Whitney test, ^x2 =^ Pearson’s Chi-squared test, ^t^ independent samples test. Statistically significant values are shown in bold.

**Table 6:** Univariate and multivariate logistic regression analysis of clinical and laboratory characteristics for prediction of disease severity.

Factor		Univariate Analysis			Multivariate Analysis		
		OR	95% CI	*p*	OR	95% CI	*p*
FMF diagnosis age	Years	0.989	0.967–1.012	0.362			
Sex	Male (RC) vs. female	1.536	0.870–2.715	0.139	-	-	-
Comorbidity index	Score	1.253	0.674–2.326	0.476			
Hb	g/dL	0.737	0.613–0.886	0.001	-	-	-
MCH	pg	0.811	0.727–0.904	<0.001	-	-	-
MCHC	g/dL	0.839	0.656–1.073	0.162	-	-	-
RDW	%	1.217	1.096–1.351	<0.001	**1.131**	**1.007–1.270**	**0.038**
NLR	High (RC) vs. low	4.273	2.370–7.703	<0.001	**-**	**-**	**-**
PLR	High (RC) vs. low	2.930	1.637–5.245	<0.001	**-**	**-**	**-**
PIV	High (RC) vs. low	7.125	3.635–13.966	<0.001	**3.133**	**1.406–6.978**	**0.005**
Sedimentation	mm/h	1.063	1.035–1.092	<0.001	**-**	**-**	**-**
CRP	mg/L	1.189	1.111–1.272	<0.001	**1.139**	**1.059–1.225**	**<0.001**
M694V homozygous mutation	Present (RC) vs. absent	2.981	1.627–5.460	<0.001	**3.714**	**1.869–7.379**	**<0.001**

FMF = familial Mediterranean fever, Hb = hemoglobin, MCH = mean corpuscular hemoglobin, MCHC = mean corpuscular hemoglobin concentration, RDW = red cell distribution width, NLR = neutrophil-to-lymphocyte ratio, PLR = platelet-to-lymphocyte ratio, PIV = pan-immune-inflammation value, CRP = C-reactive protein, RC = reference category, OR = odds ratio, CI = confidence interval. Statistically significant values are shown in bold.

## Data Availability

The data presented in this study are available on request from the corresponding author.

## References

[1] Masters S.L., Simon A., Aksentijevich I., Kastner D.L. (2009). Horror autoinflammaticus: The molecular pathophysiology of autoinflammatory disease. Annu. Rev. Immunol..

[2] Ben-Chetrit E., Touitou I. (2009). Familial Mediterranean fever in the world. Arthritis. Rheum..

[3] Chae J.J., Wood G., Masters S.L., Richard K., Park G., Smith B.J., Kastner D.L. (2006). The B30. 2 domain of pyrin, the familial Mediterranean fever protein, interacts directly with caspase-1 to modulate IL-1β production. Proc. Natl. Acad. Sci. USA.

[4] Ben-Zvi I., Livneh A. (2011). Chronic inflammation in FMF: Markers, risk factors, outcomes and therapy. Nat. Rev. Rheumatol..

[5] Parmaksız G., Noyan Z.A. (2023). Can RDW be used as a screening test for subclinical inflammation in children with FMF? Is RDW related to MEFV gene mutations?. Clin. Rheumatol..

[6] Tufan A., Lachmann H. (2020). Familial Mediterranean fever, from pathogenesis to treatment: A contemporary review. Turk. J. Med. Sci..

[7] Papa R., Lachmann H.J., Secondary A.A. (2018). Amyloidosis. Rheum. Dis. Clin. N. Am..

[8] Bilginer Y., Akpolat T., Ozen S. (2011). Renal amyloidosis in children. Pediatr. Nephrol..

[9] Erer B., Demirkaya E., Ozen S., Kallinich T. (2016). What is the best acute phase reactant for familial Mediterranean fever follow-up and its role in the prediction of complications? A systematic review. Rheumatol. Int..

[10] Tezcan M.E., Acer K.S., Şen N., Osken S., Yılmaz-Oner S. (2023). Importance of hematological markers in familial Mediterranean fever in terms of disease severity and amyloidosis. Rheumatol. Int..

[11] Keles A., Iskender D., Celik O.Y., Dagdeviren G., Iskender C., Caglar A.T., Celen S. (2021). Neutrophil-to-lymphocyte ratios in pregnant women with familial mediterranean fever. Bratisl. Lek. Listy.

[12] Yorulmaz A., Akbulut H., Taş S.A., Tıraş M., Yahya I., Peru H. (2019). Evaluation of hematological parameters in children with FMF. Clin. Rheumatol..

[13] Duksal F., Alaygut D., Güven A.S., Ekici M., Oflaz M.B., Tuncer R., Cevit Ö. (2015). Neutrophil-lymphocyte ratio in children with familial Mediterranean fever. Eur. J. Rheumatol..

[14] Ahsen A., Ulu M.S., Yuksel S., Demir K., Uysal M., Erdogan M., Acartürk G. (2013). As a new inflammatory marker for familial Mediterranean fever: Neutrophil-to-lymphocyte ratio. Inflammation.

[15] Ozer S., Yılmaz R., Sönmezgöz E., Karaaslan E., Taşkın S., Bütün İ., Demir O. (2015). Simple markers for subclinical inflammation in patients with Familial Mediterranean Fever. Med. Sci. Monit..

[16] Kelesoglu F.M., Aygun E., Okumus N.K., Ersoy A., Karapınar E., Saglam N., Aydın N.G., Senay B.B., Gonultas S., Sarisik E. (2016). Evaluation of subclinical inflammation in familial Mediterranean fever patients: Relations with mutation types and at-tack status: A retrospective study. Clin. Rheumatol..

[17] Fucà G., Guarini V., Antoniotti C., Morano F., Moretto R., Corallo S., Marmorino F., Lonardi S., Rimassa L., Sar-tore-Bianchi A. (2020). The Pan-Immune-Inflammation Value is a new prognostic biomarker in metastatic colorectal cancer: Results from a pooled-analysis of the Valentino and TRIBE first-line trials. Br. J. Cancer.

[18] Sahin A.B., Cubukcu E., Ocak B., Deligönül A., Orhan S.O., Tolunay S., Gokgoz M.S., Cetintas S., Yarbas G., Senol K. (2021). Low pan-immune-inflammation-value predicts better chemotherapy response and survival in breast cancer patients treated with neoadjuvant chemotherapy. Sci. Rep..

[19] Lee L.E., Ahn S.S., Pyo J.Y., Song J.J., Park Y.B., Lee S.W. (2021). Pan-immune-inflammation value at diagnosis independently predicts all-cause mortality in patients with antineutrophil cytoplasmic antibody-associated vasculitis. Clin. Exp. Rheumatol..

[20] Livneh A., Langevitz P., Zemer D., Kees S., Lidar T., Migdal A., Padeh S., Pras M. (1997). Criteria for the diagnosis of familial Mediterranean fever. Arthritis. Rheum..

[21] Levin A., Stevens P.E. (2013). Summary of recommendation statements. Kidney. Int. Suppl..

[22] Charlson M.E., Pompei P., Ales K.L., MacKenzie C.R. (1987). A new method of classifying prognostic comorbidity in longitudinal studies: Development and validation. J. Chronic. Dis..

[23] Demirkaya E., Acikel C., Hashkes P., Gattorno M., Gul A., Ozdogan H., Turker T., Karadag O., Livneh A., Ben-Chetrit E. (2016). Development and initial validation of international severity scoring system for familial Mediterranean fever (ISSF). Ann. Rheum. Dis..

[24] Mukhin N.A., Kozlovskaya L.V., Bogdanova M.V., Moiseev S.V., Simonyan A.K. (2015). Predictors of AA amyloidosis in familial Mediterranean fever. Rheumatol. Int..

[25] Sönmez H.E., Esmeray P., Batu E.D., Arıcı Z.S., Demir S., Sağ E., Özen S., Bilginer Y. (2019). Is age associated with disease severity and compliance to treatment in children with familial Mediterranean fever?. Rheumatol. Int..

[26] Kasifoglu T., Bilge S.Y., Sari I., Solmaz D., Senel S., Emmungil H., Kilic L., Oner S.Y., Yildiz F., Yilmaz S. (2014). Amyloidosis and its related factors in Turkish patients with familial Mediterranean fever: A multicentre study. Rheumatology.

[27] Varan O., Kucuk H., Babaoglu H., Tecer D., Atas N., Salman R.B., Satıs H., Ozturk M.A., Haznedaroglu S., Goker B. (2019). Chronic inflammation in adult familial Mediterranean fever patients: Underlying causes and association with amyloidosis. Scand. J. Rheumatol..

[28] Bas B., Sayarlioglu H., Yarar Z., Dilek M., Arik N., Sayarlioglu M. (2023). Investigation of the relationship between disease severity and development of amyloidosis and genetic mutation in FMF disease. Ir. J. Med. Sci..

[29] Tunca M., Ozdogan H., Kasapcopur O., Ozdogan H., Kasapcopur O., Yalcinkaya F., Tutar E., Ozen S., Topaloglu R., Yilmaz E. (2005). Familial Mediterranean fever (FMF) in Turkey: Results of a nationwide multicenter study. Medicine.

[30] Yasar Bilge N.S., Sari I., Solmaz D., Senel S., Emmungil H., Kilic L., Oner S.Y., Yildiz F., Yilmaz S., Bozkirli D.E. (2018). Com-parison of early versus late onset familial Mediterranean fever. Int. J. Rheum. Dis..

[31] Uslu A.U., Deveci K., Korkmaz S., Aydın B., Senel S., Sancakdar E., Sencan M. (2013). Is neutrophil/lymphocyte ratio associated with subclinical inflammation and amyloidosis in patients with familial Mediterranean fever?. Biomed. Res. Int..

[32] Sannan N.S. (2023). Assessment of aggregate index of systemic inflammation and systemic inflammatory response index in dry age-related macular degeneration: A retrospective study. Front. Med..

[33] Mangalesh S., Dudani S., Mahesh N.K. (2024). Development of a Novel Inflammatory Index to Predict Coronary Artery Disease Severity in Patients with Acute Coronary Syndrome. Angiology.

[34] Tutan D., Doğan A.G. (2023). Pan-Immune-Inflammation Index as a Biomarker for Rheumatoid Arthritis Progression and Diagnosis. Cureus.

[35] Marnell L., Mold C., Du Clos T.W. (2005). C-reactive protein: Ligands, receptors and role in inflammation. Clin. Immunol..

[36] (1997). Consortium IF Ancient missense mutations in a new member of the RoRet gene family are likely to cause familial Mediterranean fever. Cell.

[37] Kunt S.S., Aydın F., Çakar N., Özdel S., Yalçınkaya F., Özçakar Z.B. (2020). The effect of genotype on musculoskeletal complaints in patients with familial Mediterranean fever. Postgrad. Med..

[38] Grossman C., Kassel Y., Livneh A., Ben-Zvi I. (2019). Familial Mediterranean fever (FMF) phenotype in patients homozygous to the MEFV M694V mutation. Eur. J. Med. Genet..

[39] Barut K., Sahin S., Adrovic A., Sinoplu A.B., Yucel G., Pamuk G., Aydın A.K., Dasdemir S., Turanlı E.T., Buyru N. (2018). Fa-milial Mediterranean fever in childhood: A single-center experience. Rheumatol. Int..

[40] Zahorec R. (2001). Ratio of neutrophil to lymphocyte counts-rapid and simple parameter of systemic inflammation and stress in critically ill. Bratisl. Lek. Listy.

[41] Pierce C.N., Larson D.F. (2005). Inflammatory cytokine inhibition of erythropoiesis in patients implanted with a mechanical circulatory assist device. Perfusion.

[42] Vayá A., Alis R., Hernández J.-L., Calvo J., Micó L., Romagnoli M., Ricarte J.M. (2013). RDW in patients with systemic lupus erythematosus. Influence of anaemia and inflammatory markers. Clin. Hemorheol. Microcirc..

[43] Vayá A., Rivera L., Todolí J., Hernandez J.H., Laiz B., Ricart J.M. (2014). Haematological, biochemical and inflammatory parameters in inactive Behçet’s disease. Its association with red blood cell distribution width. Clin. Hemorheol. Microcirc..

[44] Rodríguez-Carrio J., Alperi-López M., López P., Alonso-Castro S., Ballina-García F.J., Suárez A. (2015). Red cell distribution width is associated with cardiovascular risk and disease parameters in rheumatoid arthritis. Rheumatology.

[45] Yildirim C.G., Gul O., Kesici-Metin F., Gokalp I., Sayarlıoglu M. (2014). Evaluation of the mean platelet volume and red cell dis-tribution width in FMF: Are they related to subclinical inflammation or not?. Int. J. Chronic. Dis..

